# Subfoveal Choroidal Thickness and Glaucoma. The Beijing Eye Study 2011

**DOI:** 10.1371/journal.pone.0107321

**Published:** 2014-09-11

**Authors:** Ya Xing Wang, Liang Xu, Lei Shao, Ya Qin Zhang, Hua Yang, Jin Da Wang, Jost B. Jonas, Wen Bin Wei

**Affiliations:** 1 Beijing Institute of Ophthalmology, Beijing Tongren Hospital, Capital Medical University, Beijing, China; 2 Department of Ophthalmology, Medical Faculty Mannheim of the Ruprecht-Karls-University, Heidelberg, Germany; 3 Beijing Tongren Eye Center, Beijing Tongren Hospital, Capital Medical University, Beijing, China; Centre for Eye Research Australia, Australia

## Abstract

**Purpose:**

To examine subfoveal choroidal thickness (SFCT) in eyes with glaucoma, using enhanced depth imaging spectral domain optical coherence tomography.

**Methods:**

The population-based Beijing Eye Study 2011 included 3468 individuals with a mean age of 64.6±9.8 years (range: 50–93 years). A detailed ophthalmic examination was performed including spectral-domain optical coherence tomography (SD-OCT) with enhanced depth imaging for measurement of SFCT, and assessment of fundus photographs for presence of glaucoma. In addition, the group of patients with chronic angle-closure glaucoma (ACG) from the Beijing Eye Study (n = 37) was merged with a group of patients with chronic ACG from the Tongren hospital (n = 52).

**Results:**

Assessments of SFCT and glaucoma were available for 3232 (93.2%) subjects. After adjusting for age, axial length, gender, anterior chamber and lens thickness, SFCT was not significantly associated with presence of glaucoma (*P* = 0.08; regression coefficient B:−15.7). As a corollary, in logistic regression analysis with adjustment for age, axial length and intraocular pressure, presence of glaucoma was not significantly associated with SFCT (*P* = 0.20). If only open-angle glaucoma was considered, multivariate analysis revealed no significant association between SFCT and presence of open-angle glaucoma (*P* = 0.44). As a corollary, in logistic regression analysis, open-angle glaucoma was not significantly associated with SFCT (*P* = 0.91). In a similar manner if only ACG was taken into account, SFCT was not significantly associated with the presence of ACG (*P* = 0.27) in multivariate analysis. As a corollary in binary regression analysis, presence of ACG was not significantly associated with SFCT (*P* = 0.27).

**Conclusions:**

In multivariate analysis with adjustment for age, axial length, gender, anterior chamber and lens thickness, neither OAG nor ACG was associated with an abnormal SFCT.

## Introduction

Glaucomatous optic neuropathy is characterized by the loss of retinal ganglion cell axons, leading to marked morphological changes in the optic nerve head and the inner retinal layers [1]. Additional glaucoma-related changes in the deep retinal layers and the choroid were suggested by few histomorphometric studies and by some recent clinical studies, while other investigations contradicted the notion of glaucomatous changes at the level of the photoreceptors, retinal pigment epithelium and choroid [2]–[11]. Investigations focused on patients with acute angle-closure glaucoma suggested that eyes with angle-closure glaucoma have an abnormally thick choroid. It has been discussed, that this choroidal abnormality may be involved in the pathogenesis of angle-closure glaucoma [12], [13]. Other studies did not find differences in subfoveal choroidal thickness (SFCT) between eyes with open-angle glaucoma and control eyes or assessed the choroidal thickness in the parapapillary or perifoveal region [14]–[26]. Since the previous studies were hospital-based investigations with the potential risk of a bias due to the referral of patients or since these studies measured the ocular dimensions in fixed eyes with postmortal swelling and fixation induced shrinkage, it remains unclear whether patients with open-angle glaucoma and patients with angle-closure glaucoma show abnormalities in choroidal thickness. Knowledge about the choroidal thickness in glaucoma would help in the discussion whether glaucoma is additionally associated with changes in the deep retinal layers and the choroid. It may also be of interest for the diagnosis of glaucoma, since deep retinal changes show a different pattern in the psychophysical examinations such as perimetry and color vision testing, and since glaucoma related changes in the deep retinal layers and in the choroid could be visualized and analyzed by optical coherence tomography (OCT). We therefore planned and prospectively performed this study to measure the choroidal thickness in patients with glaucoma and non-glaucomatous subjects in a population-based setting. We defined glaucoma by the appearance of the optic nerve head and graduated the amount of glaucomatous optic nerve damage by the thickness of the retinal nerve fiber layer.

## Methods

### Ethics Statement

The Medical Ethics Committee of the Beijing Tongren Hospital approved the study protocol of the Beijing Eye Study and the protocol for including the additional patients examined in the Beijing Tongren hospital and all participants gave informed written consent, according to the Declaration of Helsinki.

The Beijing Eye Study 2011 is a population-based cross-sectional study in Northern China [27], [28]. It was carried out in 5 communities in the urban district of Haidian in the North of Central Beijing and in 3 communities in the village area of Yufa of the Daxing District south of Beijing. The only eligibility criterion for inclusion into the study was an age of 50+ years. In 2011, the 8 communities had a total population of 4403 individuals aged 50 years or older. In total, 3468 individuals (1963 (56.6%) women) participated in the eye examination, corresponding to an overall response rate of 78.8%. The study was divided into a rural part (1633 (47.1%) subjects; 943 (57.7%) women) and an urban part (1835 (52.9%) subjects; 1020 (55.6%) women). The mean age was 64.6±9.8 years (median, 64 years; range, 50–93 years). All study participants underwent an interview with standardized questions on their family status, level of education, physical activity, and known major systemic diseases. The ophthalmic examination included measurement of presenting visual acuity, uncorrected visual acuity, and best corrected visual acuity, tonometry, slit lamp examination of the anterior and posterior segment of the eye, biometry for measurement of the anterior corneal curvature, central corneal thickness, anterior chamber depth, lens thickness and axial length, and digital photography of the cornea, lens, macula and optic disc (fundus camera Type CR6-45NM; Canon Inc, Tokyo, Japan). The retinal nerve fiber thickness was measured by spectral domain OCT (Spectralis, Heidelberg Engineering Co., Heidelberg, Germany).

SFCT was measured using spectral domain optical coherence tomography (SD-OCT; Spectralis, Wavelength: 870 nm; Heidelberg Engineering Co., Heidelberg, Germany) with enhanced depth imaging (EDI-OCT) modality after pupil dilation [28], [29]. Seven sections, each comprising 100 averaged scans, were obtained in an angle of 5°–30° rectangle centered onto the fovea. The horizontal section running through the center of the fovea was selected for further analysis. Subfoveal choroidal thickness was defined as the vertical distance from the hyperreflective line of the Bruch's membrane to the hyperreflective line of the inner surface of the sclera. The measurements were performed using the Heidelberg Eye Explorer software (version 5.3.3.0; Heidelberg Engineering Co., Heidelberg, Germany). Only the right eye of each study participant was measured. The images were taken by one technician (CXC) and the images were assessed by two ophthalmologists (LS, KFD). The reproducibility of the technique was previously examined and revealed a high reproducibility (Bland-Altman plot with 1.9% (61/3233) points outside the 95% limits of agreement; intra-class coefficient: 1.00; mean coefficient of variation: 0.85%±1.48%) [30].

Examining the digital photographs of the optic disc and macula, glaucoma was defined by a glaucomatous appearance of the optic disc. The optic nerve head was glaucomatous (1) if the inferior-superior-nasal-temporal (ISNT)-rule of the neuroretinal rim shape was not fulfilled in early glaucoma and in eyes with a normally shaped optic disc (it included a notch in the neuroretinal rim in the temporal inferior region and/or the temporal superior region); or (2) if the neuroretinal rim was too small in relation to the size of the optic disc. For all situations, the retinal nerve fiber layer had to show a localized and/or diffuse loss. The assessment of the optic disc photographs was carried in a masked manner without knowledge of intraocular pressure. Each photograph of a glaucomatous optic disc was independently adjudicated by three senior graders (LX, YXW, JBJ). The whole glaucoma group was differentiated into subjects with open-angle glaucoma and with primary angle closure glaucoma. Open-angle glaucoma was characterized by an open anterior chamber angle, in addition to a normal depth of the anterior chamber as assessed by slit lamp biomicroscopy. In angle-closure glaucoma, the anterior chamber angle was occluded or occludable. Using the definition by Foster and colleagues, the anterior chamber angle was defined as occludable, if ≥270° of the posterior trabecular meshwork could not be seen upon gonioscopy [31]. In addition, other features for angle-closure glaucoma were iris whirling and glaukomflecken in the anterior subcapsular lens region, in combination with a narrow anterior chamber angle.

For a subgroup of study participants, optical coherence tomography (iTVue SD-OCT; Optovue Inc. Fremont, CA, U.S.A.) of the optic nerve head was performed and the vertical cup/disc diameter ratio among other optic disc parameters was measured. For this subgroup, we additionally assessed the presence of glaucoma using criteria of the International Society of Geographic and Epidemiological Ophthalmology ISGEO [31].

Statistical analysis was performed using a commercially available statistical software package (SPSS for Windows, version 20.0, IBM-SPSS, Chicago, IL). In a first step, we examined the mean values (presented as mean ± standard deviation) of SFCT. In a second step, we compared the SFCT between the study groups. In a third step, we performed a multivariate linear regression analysis, with SFCT as dependent parameter and those parameters as independent parameters which had previously been shown to be associated with SFCT.^28^ Odds ratios (OR) and 95% confidence intervals (CI) were presented. All *P*-values were 2-sided and were considered statistically significant when the values were less than 0.05.

## Results

Out of the 3468 participants, measurements of the SFCT and assessments for glaucoma were available for 3232 (93.2%) subjects (1817 (56.2%) women). The mean age was 64.3±9.6 years (median: 63 years; range: 50 to 93 years), mean refractive error (spherical equivalent) was −0.18±1.98 diopters (median: 0.25 diopters; range: −20.0 to +7.00 diopters), and mean axial length was 23.24±1.11 mm (median: 23.13 mm; range: 18.96–30.88 mm). The group of subjects without measurements of the SFCT and without assessment of glaucoma as compared with the group of subjects with both examinations was significantly (*P*<0.001) older (69.5±10.9 years versus 64.3±9.6 years) and was more myopic (−1.57±4.47 diopters versus −0.18±1.98 diopters; *P* = 0.002) and did not vary significantly in gender (*P* = 0.09).

Glaucomatous optic neuropathy was detected in 128 (4.0%) patients. The glaucoma group could be further subdivided into subjects with open-angle glaucoma (n = 90 (2.8%)), primary angle-closure glaucoma (n = 37 (1.1%) and secondary angle-closure glaucoma (n = 1 (0.03%)). The glaucoma group as a whole and differentiated into the open-angle glaucoma group and the primary angle-closure glaucoma group as compared with the control group was significantly (*P*<0.001) older (Table 1). Axial length was significantly longer in the total glaucoma group (*P* = 0.01) and in the open-angle glaucoma group (*P* = 0.001) than in the control group, while the angle-closure glaucoma and the control group did not differ significantly (*P* = 0.09) in axial length (Table 1). Mean retinal nerve fiber layer thickness was significantly thinner in the glaucoma group than in the non-glaucomatous group (101.5±11.6 µm versus 85.5±17.1 µm; *P*<0.001).

**Table 1:** Demographic parameters, ocular parameters and subfoveal choroidal thickness (mean ± standard deviation) in groups of patients with different types of glaucoma and the remaining control group in the Beijing Eye Study 2011.

	Total Glaucoma Group	Open-Angle Glaucoma	Angle Closure	Control Group	*P*-Value (1)	*P*-Value (2)	*P*-Value (3)
n	128	90	37	3104			
Age	70.9±9.8	70.0±10.1	72.9±8.8	63.9±9.5	<0.001	<0.001	<0.001
Refractive Error	−0.50±2.24	−0.67±2.41	−0.13±2.14	−0.16±1.96	0.11	0.053	0.91
Axial Length	23.5±1.3	23.8±1.4	23.0±0.8	23.2±1.1	0.01	0.001	0.09
SFCT	201.4±02.4	210.1±104.7	184.2±93,6	255.9±107.0	<0.001	<0.001	<0.001

P-Value (1): Statistical significance of the difference between the total glaucoma group and the control group.

P-Value (2): Statistical significance of the difference between the open-angle glaucoma group and the control group.

P-Value (3): Statistical significance of the difference between the angle-closure glaucoma group and the control group.

One glaucoma patient had secondary angle-closure glaucoma.

In univariate analysis, mean SFCT was significantly (*P*<0.001) thinner in the total glaucoma group as a whole (201±102 µm (median: 188 µm; range: 9 µm to 537 µm)), and separated into the open-angle glaucoma group (210±105 µm (median: 210 µm; range: 9 µm to 537 µm)), and into the angle-closure glaucoma group (184±94 µm (median: 155 µm; range: 49 µm to 399 µm)) than in the control group (256±107 µm (median: 252 µm; range: 8 µm to 854 µm)) (Table 1).

Since SFCT has been shown to be associated with younger age, shorter axial length, male gender, deeper anterior chamber, thicker lens and presence of diabetes mellitus in the study population of the Beijing Eye Study [28], [32], we performed a multivariate analysis with SFCT as dependent variable and age, axial length, gender, anterior chamber depth, lens thickness, presence of diabetes mellitus and presence of glaucoma as independent variables. It revealed that SFCT was significantly associated with younger age (*P*<0.001), shorter axial length (*P*<0.001), male gender (*P*<0.001), deeper anterior chamber (*P*<0.001), larger lens thickness (*P*<0.001) and presence of diabetes mellitus (*P* = 0.01), while the presence of glaucoma (*P* = 0.07) was not significantly associated (Table 2). Analysis of collinearity revealed that the variance inflation factors were <1.90 for all parameters included into the analysis. The variance inflation factor for the presence of glaucoma was 1.02. If intraocular pressure was added to the list of independent parameters, neither glaucoma (*P* = 0.09) nor intraocular pressure (*P* = 0.94) were significantly associated with SFCT. If a logistic regression analysis was performed with the presence of glaucoma as dependent variable, and age, axial length, intraocular pressure (factors which in a previous study were significantly associated with the prevalence of glaucoma) and SFCT as independent variables [27], presence of glaucoma was significantly associated with older age (*P*<0.001; regression coefficient B: 0.07; OR: 1.07 (95%CI: 1.05, 1.09) and higher intraocular pressure (*P* = 0.004; B: 0.13; OR: 1.14 (95%CI: 1.07, 1.21), while axial length (*P* = 0.08; B: 0.15; OR: 1.16 (95%CI: 0.99, 1.36) and SFCT (*P* = 0.20; B: −0.001; OR: 1.00 (95%CI: 1.00, 1.00) were not significantly associated with glaucoma.

**Table 2:** Associations (multivariate analysis) between subfoveal choroidal thickness, general parameters and glaucoma in the Beijing Eye Study 2011.

Parameter	*P*-Value	Standardized Coefficient Beta	Regression Coefficient B	95% Confidence Intervals for B
Age (Years)	<0.001	−0.41	−4.63	−5.02, −4.24
Axial Length (mm)	<0.001	−0.38	−36.8	−40.4, −33.1
Gender (Men/Women)	<0.001	−0.15	−32.7	−39.6, −25.8
Anterior Chamber Depth (mm)	<0.001	−0.08	26.4	13.5, 39.4
Lens Thickness (mm)	<0.001	0.08	25.0	13.0, 37.0
Diabetes Mellitus	0.01	0.04	11.7	2.43, 21.0
Glaucoma	0.07	−0.03	−16.7	−34.8,1.32

Within the total glaucoma group, retinal nerve fiber layer thickness was significantly (*P* = 0.003) associated with SFCT in univariate analysis. If age was added to the list of independent parameters, only age was correlated with SFCT (*P*<0.001; correlation coefficient: −0.46), while SFCT was not related with retinal nerve fiber layer thickness (*P* = 0.65). A similar result was obtained, if additionally axial length was added to the multivariate analysis. If the association was adjusted for age, axial length, gender, anterior chamber depth and lens thickness, the relationship between SFCT and retinal nerve fiber layer thickness again was not statistically significant (*P* = 0.62). If the whole glaucoma group was split up into the open-angle glaucoma subgroup and the angle-glaucoma subgroup, the SFCT was not significantly related with retinal nerve fiber layer thickness, neither in the open-angle glaucoma subgroup (*P* = 0.69) nor in the angle-glaucoma subgroup (*P* = 0.85).

Optical coherence tomography of the optic disc was performed on 1587 (49.1% from the study population) subjects with a mean age of 66.1±9.9 years and a mean axial length of 23.39±1.12 mm. Out of these 1587 subjects, 42 subjects had a vertical cup/disc ratio of 0.91 (i.e. 97.5 percentile) or larger, 29 subjects had history of glaucoma treatment, and 59 subjects had an inferior or superior rim area less than the 97.5% percentile. Altogether (including the overlapping cases), 97 (6.1%) subjects fulfilled at least one of the criteria. Using this glaucoma definition and re-calculating the multivariable analysis revealed that SFCT was significantly associated with younger age (*P*<0.001), shorter axial length (*P*<0.001), and male gender (*P*<0.001). It was not significantly associated with anterior chamber (*P* = 0.08), lens thickness (*P* = 0.11) presence of diabetes mellitus (*P* = 0.86) nor presence of glaucoma (*P* = 0.42).

If only the group of patients with open-angle glaucoma was considered, SFCT was significantly associated with younger age (*P*<0.001), shorter axial length (*P*<0.001), male gender (*P*<0.001), deeper anterior chamber (*P*<0.001), larger lens thickness (*P*<0.001) and presence of diabetes mellitus (*P* = 0.01), while the presence of open-angle glaucoma (*P* = 0.41) was not significantly associated (Table 3). If intraocular pressure was added to the list of independent parameters, neither open-angle glaucoma (*P* = 0.40) nor intraocular pressure (*P* = 0.99) were significantly associated with SFCT. If a logistic regression analysis was performed with the presence of open-angle glaucoma as dependent variable, and age, axial length and intraocular pressure and SFCT as independent variables, presence of open-angle glaucoma was significantly associated with older age (*P*<0.001; regression coefficient B: 0.07; OR: 1.07 (95%CI: 1.04, 1.09), higher intraocular pressure (*P* = 0.004; B: 0.12; OR: 1.13 (95%CI: 1.05, 1.21) and longer axial length (*P* = 0.001; B: 0.30; OR: 1.35 (95%CI: 1.13, 1.61), while SFCT (*P* = 0.91) was not significantly associated with open-angle glaucoma. Within the group with open-angle glaucoma, 7 (8%) eyes had undergone anti-glaucomatous filtering surgery one or more years prior to inclusion into the study. The subgroup with glaucoma surgery and the subgroup without glaucoma surgery did not vary significantly in SFCT (262±96 µm versus 206±105 µm; *P* = 0.18) or in age (71.9±10.3 years versus 70.1±10.1 years; *P* = 0.81), while axial length was significantly shorter in the surgical subgroup (22.5±1.1 mm versus 23.9±1.4 mm; *P* = 0.01). After adjusting for axial length, SFCT was again not significantly associated with previous glaucoma surgery.

**Table 3:** Associations (multivariate analysis) between subfoveal choroidal thickness, general parameters and open-angle glaucoma in the Beijing Eye Study 2011.

Parameter	*P*-Value	Standardized Coefficient Beta	Regression Coefficient B	95% Confidence Intervals for B
Age (Years)	<0.001	−0.41	−4.65	−5.04, −4.26
Axial Length (mm)	<0.001	−0.38	−36.9	−40.5, −33.2
Gender (Men/Women)	<0.001	−0.15	−32.8	−39.7, −25.9
Anterior Chamber Depth (mm)	<0.001	−0.08	25.1	12.0, 38.1
Lens Thickness (mm)	<0.001	0.08	24.7	12.7, 36.7
Diabetes Mellitus	0.01	0.04	11.6	2.34, 20.9
Open-Angle Glaucoma	0.41	−0.01	−9.1	−30.5,12.4

If only the group of patients with primary angle-closure glaucoma was considered, thicker SFCT was significantly associated with younger age (*P*<0.001), shorter axial length (*P*<0.001), male gender (*P*<0.001), deeper anterior chamber (*P*<0.001), larger lens thickness (*P*<0.001), presence of diabetes mellitus (*P* = 0.01), and the absence of angle-closure glaucoma (*P* = 0.044) (Table 4). If intraocular pressure was added to the list of independent parameters, neither angle-closure glaucoma (*P* = 0.07) nor intraocular pressure (*P* = 0.97) were significantly associated with SFCT. If a logistic regression analysis was performed with the presence of angle-closure glaucoma as dependent variable, and age, axial length and intraocular pressure, and SFCT as independent variables, presence of angle-closure glaucoma was significantly associated with older age (*P*<0.001; regression coefficient B: 0.08; OR: 1.08 (95%CI: 1.04, 1.13), higher intraocular pressure (*P* = 0.004; B: 0.15; OR: 1.16 (95%CI: 1.05, 1.29), shorter axial length (*P* = 0.048; B: −0.34; OR: 0.71 (95%CI: 0.50, 1.00), and thinner SFCT (*P* = 0.04; B: −0.01; OR: 0.995 (95%CI: 0.991, 1.00). Within the group with angle-closure glaucoma, 8 (22%) eyes had undergone anti-glaucomatous filtering surgery one or more years prior to inclusion into the study. The subgroup with glaucoma surgery and the subgroup without glaucoma surgery did not vary significantly in SFCT (185±98 µm versus 184±94 µm; *P* = 0.98) or in age (71.9±10.3 years versus 73.2±8.4 years; *P* = 0.74) or in axial length 22.6±0.9 mm versus 23.1±0.7 mm; *P* = 0.12).

**Table 4:** Associations (multivariate analysis) between subfoveal choroidal thickness, general parameters and angle-closure glaucoma in the Beijing Eye Study 2011.

Parameter	*P*-Value	Standardized Coefficient Beta	Regression Coefficient B	95% Confidence Intervals for B
Age (Years)	<0.001	−0.41	−4.64	−5.03, −4.25
Axial Length (mm)	<0.001	−0.38	−36.8	−40.5, −33.2
Gender (Men/Women)	<0.001	−0.15	−32.6	−39.5, −25.8
Anterior Chamber Depth (mm)	<0.001	−0.08	24.8	12.8, 36.8
Lens Thickness (mm)	<0.001	0.08	24.8	12.8, 36.8
Diabetes Mellitus	0.01	0.04	11.9	2.65, 21.2
Angle-Closure Glaucoma	0.044	−0.03	−33.4	−66.0,−0.89

Since the number of individuals with primary angle-closure glaucoma was relatively small (n = 37), we added a second group of patients (n = 52) with chronic primary angle-closure glaucoma who had been diagnosed and treated in the Beijing Tongren hospital. There had been no known glaucoma attack in at least two months preceding the examination. As the participants of the Beijing Eye Study, the patients with chronic angle-closure glaucoma from the Tongren hospital had undergone an ophthalmological examination including spectral-domain OCT with enhanced depth imaging to visualize and measure the SFCT. Mean age was 62.3±7.2 years (median: 62 years; range: 44–76 years). If this hospital-based group of patients with angle-closure glaucoma was merged with the population-based group of patients with angle-closure glaucoma, SFCT was significantly thinner in the chronic angle-closure glaucoma group than in the non-glaucomatous group (222±90 µm versus 256±107 µm; *P*<0.001). In multivariate analysis, with SFCT as a dependent variable and refractive error, age and presence of chronic angle-closure glaucoma as independent variables, thinner SFCT was significantly associated with the older age (*P*<0.001; beta: −0.43; B: −4.84 (95%CI: −5.18, −4.50)) and myopic refractive error (*P*<0.001; beta: 0.29; B: 16.2 (95%CI: 14.5, 17.8)) while it was not significantly associated with the presence of chronic angle-closure glaucoma (*P* = 0.27; beta: −0.02; B: −13.1 (95%CI: −38.8, 11.0)). In binary regression analysis, presence of chronic angle-closure glaucoma was significantly associated with older age (*P*<0.001; OR: 1.08 (95%CI: 1.05, 1.12)) but not with SFCT (*P* = 0.27; OR: 0.998 (95%CI: 0.005, 1.001)).

To reduce the potential influence of outliers, the analysis was repeated after excluding all subjects with a SFCT measurement outside of the 95% confidence intervals (i.e. smaller than 69 µm or larger than 464 µm). It revealed that SFCT was significantly associated with younger age (*P*<0.001; standardized coefficient beta: −0.40; regression coefficient B: −4.04 (95%CI: −4.39, −3.68), shorter axial length (*P*<0.001; beta: −0.36; B: −32.9 (95%CI: −36.4, −29.4)), male gender (*P*<0.001; beta: −0.14; B: −25.6 (95%CI: −31.9, −19.3)), deeper anterior chamber (*P*<0.001; beta: −0.08; B: 21.4 (95%CI: 9.5, 33.2)) and larger lens thickness (*P*<0.001; beta: 0.08; B: 22.4 (95%CI: 11.6, 33.3)), while presence of diabetes mellitus and presence of glaucoma (*P* = 0.08; beta: −0.03; B: −14.8 (95%CI: −31.4, 1.8)) were not significantly associated. In a similar manner, open-angle glaucoma (*P* = 0.36) nor angle-closure glaucoma (*P* = 0.08) were significantly associated with SFCT.

## Discussion

Since the landmark study by Spaide and colleagues on the development of enhanced depth imaging by optical coherence tomography [29], an increasing number of studies have addressed choroidal thickness in normal eyes, the factors associated with choroidal thickness in normal eyes, and associations of choroidal thickness with various retinal and retino-choroidal disorders [33]–[35]. These studies have revealed that the mean subfoveal choroidal thickness (SFCT) in normal eyes is approximately 250 µm in a population with a mean age of 65 years [28]; that it shows a huge range between values as low as 8 µm and values as large as 854 µm, that it decreases with age by 4 µm per year of age and with increasing myopia by 15 µm per diopter of myopia; and that it is additionally associated with male gender, a deeper anterior chamber and thicker lens [28]. Clinical studies also showed that patients with central serous chorioretinopathy have a thickened SFCT in the affected eye as well as in the contralateral unaffected eye [33], and that patients with polypoidal vascular choroidopathy have an increased thickness of the subfoveal choroid in association with a dilatation of the large choroidal vessels [34]. In our population-based study on a relatively large study population, we found that neither patients with open-angle glaucoma nor patients with chronic angle-closure glaucoma have an abnormal SFCT in multivariate analysis with adjustment for age, axial length, gender, anterior chamber and lens thickness.

The finding that patients with open-angle glaucoma did not differ in SFCT from normal subjects agrees with previous hospital-based studies. In the study by Mwanza and colleagues, 36 eyes with unilateral advanced glaucoma as compared with the contralateral eyes with no or mild glaucoma were examined [19]. After adjusting for axial length and intraocular pressure, choroidal thickness did not differ significantly between both groups (*P* = 0.78 to 0.99). Visual field mean deviation did not correlate with choroidal thickness measurements. In a similar manner in our study, SFCT was not significantly (*P* = 0.65) related with retinal nerve fiber layer thickness as another surrogate for the amount of optic nerve damage in glaucoma. Maul et al. reported in another study that choroidal thickness did not differ among normal subjects, patients with normal-pressure glaucoma and patients with primary open-angle glaucoma [16]. SFCT was not significantly associated with glaucoma damage severity as estimated by visual field mean deviation or nerve fiber layer thickness. In the study by Rhew and coworkers, SFCT was measured in 32 patients with normal-tension glaucoma and compared with 35 normal individuals [25]. The mean SFCT in the normal individual group and the normal-pressure glaucoma patient group were 300.0±52.7 and 289.5±100.4 µm, with no significant difference between both groups (*P* = 0.60). The peripapillary choroidal thickness in healthy controls and in patients with glaucoma with focal, diffuse and sclerotic glaucomatous optic disc damages were examined by Roberts and colleagues [20]. The authors found that peripapillary choroidal thickness did not differ significantly between glaucoma patients with focal and diffuse optic disc damage as compared to control subjects, while it was approximately 30% lower in patients with sclerotic glaucomatous optic disc damage (*P* = 0.001).

With respect to choroidal thickness in angle-closure glaucoma, Arora and colleagues recently examined 106 patients with open-angle and 79 patients with angle-closure with or without glaucoma and 40 control subjects [12]. Choroidal thickness was significantly greater in the angle-closure glaucoma group than in the open-angle glaucoma group and the normal subjects (*P*≤0.05), but there was no significant difference between the open-angle glaucoma group and the normal subjects. After adjusting for age, axial length, intraocular pressure, central corneal thickness, choroidal thickness was significantly greater in the angle-closure glaucoma group than either in the normal or the open-angle glaucoma group (*P* = 0.003 and *P* = 0.03, respectively). Interestingly, the severity of glaucomatous optic nerve damage as measured by cup/disc ratio cup-to-disc ratio or visual field mean deviation was not significantly associated with choroidal thickness. In another investigation, Arora and coworkers found that a significant increase in choroidal thickness and a decrease in anterior chamber depth when a water drinking test was performed in eyes with anterior chamber angle closure as compared to eyes with open anterior chamber angles [13]. While the study by Arora and coworkers agrees with our study in that patients with open-angle glaucoma and normal subjects do not differ significantly in choroidal thickness, both studies disagree on the results for angle-closure: While we found, that eyes with chronic angle-closure glaucoma have a normal SFCT, the choroid was abnormally thick in the subjects with angle-closure in the study by Arora. In another recent study on fellow eyes of 44 patients with unilateral acute primary angle-closure in the contralateral eyes, Zhou and colleagues found that the fellow eyes had a thicker choroid than a group of control eyes after adjusting for age, axial length, and gender [36]. The reasons for the discrepancies between the studies have remained unclear. A potential cause for the discrepancy between the studies could be a difference between acute angle-closure glaucoma and chronic angle-closure glaucoma. Most of the preceding studies showing an association between abnormally thick SFCT and angle-closure glaucoma included patients shortly after an acute angle-closure glaucoma attack. There may be the possibility that shortly after a high intraocular pressure period such as after an acute glaucoma attack, the choroidal vessels show a reactive dilatation. It could lead to an overestimation of choroidal thickness in these eyes with acute angle-closure glaucoma as compared to eyes with chronic angle-closure glaucoma.

Interestingly, a histomorphometric study on human enucleated eyes with absolute secondary angle-closure glaucoma revealed that the choroid was significantly thinner in the glaucoma group than in the control group [2]. In a similar manner, Hayreh and colleagues reported on a positive correlation between the optic nerve head damage and atrophic changes in the temporal peripapillary choroid in monkeys with experimental high-pressure glaucoma [5]. Spraul *et al.* found that eyes with advanced glaucomatous damage after long standing primary open-angle glaucoma exhibited a decreased density of capillaries of the choriocapillaris and decreased density of large choroidal vessels [7]. In a histologic study by Yin and coworkers, a reduced choroidal thickness was found in enucleated human eyes with primary open-angle glaucoma with a loss of the innermost choroidal vessels [37]. Other studies reported on tissue loss in the deep retinal layers [6], [8]–[10]. These histologic studies were however not corrected for the dependence of choroidal thickness on age and axial length so that it has remained unclear whether and how their results may contribute to the current discussion.

Potential limitations of our study should be mentioned. First, a major concern in any prevalence study is nonparticipation. The Beijing Eye Study 2011 had a reasonable response rate of 78.8%, however, differences between participants and non-participants could have led to a selection artifact. Since the group of subjects without OCT measurements and assessments of the presence of glaucoma as compared to the group of subjects with these measurements was significantly (*P*<0.001) older more myopic (*P* = 0.002), one may argue that the non-participation of a part of the elderly eligible study population may have influenced the results of the investigation. Second, previous studies by Chakraborty and colleagues and others have shown a circadian (diurnal) rhythm of about 20–30 micron change in choroidal thickness measurements by OCT [38]. The participants of our study underwent the OCT examinations at various times of the day. Since these examinations were performed in a randomized manner with respect to when they were performed, it is unlikely that the reported dependence of the choroidal thickness measurement on the time of the day introduced a bias into our study. Third, choroidal thickness was examined only in the right eye of each study participant, so that inter-eye differences and their associations with inter-eye differences of other parameters could not be assessed. Fourth, the definition of glaucoma applied in our study did not follow the recommendations of the International Society of Geographical and Epidemiological Ophthalmology [31]. If however, criteria of the ISGEO were applied for a subgroup of subjects, again the SFCT was not significantly (*P* = 0.42) associated with glaucoma after adjustment for age, axial length, gender, anterior chamber depth, lens thickness and presence of diabetes mellitus. Fifth, some eyes of the study group had undergone anti-glaucomatous filtering surgery. Previous studies have shown that filtering surgery can lead to a change in SFCT, i.e., a thickening. SFCT however was not thicker in the group of eyes with chronic angle-closure glaucoma than in the non-glaucomatous group. Sixth, differences between this study and previous investigations in SFCT and its associations with angle-closure glaucoma may have been due to differences in the ethnic background. The strengths of our study are its design as a population-based investigations and the relatively large number of study participants.

In conclusion, in multivariate analysis with adjustment for age, axial length, gender, anterior chamber and lens thickness, neither open-angle glaucoma nor chronic angle-closure glaucoma was associated with an abnormal SFCT.
